# Evaluation of an Early Intervention Model for Child and Adolescent Victims of Interpersonal Violence

**DOI:** 10.3390/children8100941

**Published:** 2021-10-19

**Authors:** Claudia Calvano, Elena Murray, Lea Bentz, Sascha Bos, Kathrin Reiter, Loretta Ihme, Sibylle M. Winter

**Affiliations:** 1 Department of Child and Adolescent Psychiatry, Charité—Universitätsmedizin Berlin, Corporate Member of Freie Universität Berlin, Humboldt-Universität zu Berlin and Berlin Institute of Health, Augustenburger Platz 1, 13353 Berlin, Germany; elena.murray@charite.de (E.M.); lea.bentz@charite.de (L.B.); sascha.bos@charite.de (S.B.); kathrin.reiter@charite.de (K.R.); sibylle.winter@charite.de (S.M.W.); 2 Clinical Child and Adolescent Psychology and Psychotherapy, Freie Universität Berlin, Habelschwerdter Allee 45, 14195 Berlin, Germany; 3 Center for Chronically Sick Children, Charité—Universitätsmedizin Berlin, Corporate Member of Freie Universität Berlin, Humboldt-Universität zu Berlin and Berlin Institute of Health, Augustenburger Platz 1, 13353 Berlin, Germany; loretta.ihme@charite.de

**Keywords:** children and adolescents, clinical practice, early intervention, interpersonal violence, sexual abuse

## Abstract

Only the minority of youth exposed to traumatic events receive mental health care, as trauma-informed clinical services are lacking or are poorly accessible. In order to bridge this gap, the Outpatient Trauma Clinic (OTC) was founded, an easily accessible early, short-time intervention, with onward referral to follow-up treatment. This report presents the OTC’s interventional approach and first outcome data. Using a retrospective naturalistic design, we analyzed trauma- and intervention-related data of the sample (*n* = 377, 55.4% female, mean age 10.95, SD = 4.69). Following drop-out analyses, predictors for treatment outcome were identified by logistic regression. The majority (81.9%) was suffering from posttraumatic stress disorder (PTSD) or adjustment disorders. Around one forth dropped out of treatment; these cases showed higher avoidance symptoms at presentation. In 91%, psychological symptoms improved. Experience of multiple traumatic events was the strongest predictor for poor treatment outcome (B = −0.823, SE = 0.313, OR = 0.439, 95% CI 0.238–0.811). Around two thirds were connected to follow-up treatment. The OTC realized a high retention rate, initial improvement of symptoms and referral to subsequent longer-term psychotherapeutic treatment in the majority. Further dissemination of comparable early intervention models is needed, in order to improve mental health care for this vulnerable group.

## 1. Introduction

According to epidemiological studies, around 30% of children and adolescents experience at least one form of interpersonal trauma [1,2]. In a substantial number of cases, this is associated with an increased risk for disturbances in mental and physical development [3,4,5]. Post-traumatic stress-disorder, affective and anxiety disorders as well as behavioral problems are among the most frequent trauma-related mental disorders in children and adolescents [1,6]. Besides a child’s female gender [7,8,9] and a young age at the first traumatic event [10], victims of sexual abuse [4,11] and victims of multiple trauma [12] are particularly at risk for poor prognosis.

In light of the high prevalence, poor prognoses and the substantial costs for the health system [13], effective treatment is urgently needed. Comprehensive meta-analyses show that trauma-focused psychological interventions are effective for the reduction of PTSD, depression and anxiety in children and adolescents, across age groups and trauma types, in controlled and uncontrolled trials [14], with promising results for long-term effects [15]. In light of this evidence, translation of trauma-informed intervention into clinical practice is warranted. In order to attain this goal and to improve the health care situation of trauma victims, different approaches and structures of trauma care have been established across Europe in recent years, with variations due to cultural backgrounds and (health-)economic situations [16,17]. However, the countries face similar challenges in the translation of evidence-based intervention into practice and in the promotion and dissemination of trauma-informed treatment models. In Germany, counseling services, crisis intervention and outpatient clinics for victims of interpersonal violence, often affiliated with psychiatric hospitals, have been emerging [16].

Despite these efforts, there is still a discrepancy between the need, the accessibility and the availability of trauma-informed clinical services [16]. For children and adolescents, there is an especially pronounced gap in health care provision and only the minority of children and adolescents exposed to traumatic events receive adequate treatment [18,19,20,21]. Data published in 2017 illustrate the situation of trauma care for children and adolescents in Germany: Münzer et al. analyzed the health care utilization of 241 children aged 4–17 years with a history of maltreatment in Germany and point out that 65% of the sample did not receive any psychotherapeutic intervention—and among the cases who did, only a few received trauma-focused psychotherapy [19]. Barriers for seeking and accessing mental health care for affected children and adolescents are by large part structural, e.g., lack of coordination and integration of services, service costs and long waiting times [22]. In a qualitative study [18], internet posts by young people in online trauma forums were analyzed. Their data suggest that structural barriers are not only at work when seeking care, but also at the end of treatment, when guidance for the patients through the pathways within the health care system seems warranted [18].

The Berlin Outpatient Trauma Clinic (OTC) for children and adolescents aims to bridge the void in both the availability of early trauma-informed care and in the connection to longer-term psychological intervention, if needed. In line with the approaches outlined in [16], the Berlin Outpatient Trauma Clinic is affiliated with the Department for Child and Adolescent Psychiatry, Charité—Universitätsmedizin Berlin, Germany. It was founded in 2012 in order to establish an easily accessible, early and specialized outpatient treatment setting for child and adolescent victims of interpersonal violence. Within a short-term early intervention setting, the OTC offers clinical assessment, stabilization and first psychotherapeutic support, as well as an onward referral to longer-term follow-up treatment. The OTC is the first treatment center the affected families consult after the traumatic event. Besides self-referrals by the families, a variety of institutions like the pediatric clinic, social services or the police refer the families to the clinic. The treatment in the OTC is independent of health insurance status as it is funded by the Crime Victims’ Compensation Act, which defines the entitlement for compensation for victims of a violent crime [23]. So far, the OTC represents the only trauma-specific early intervention clinic for both children and adolescents in Berlin, financed by the Berlin Senate.

While the need for further implementation of trauma care for children and adolescents is consistently underlined in the literature [18,19,20,21], and approaches have been emerging [16], up to now, little is known about the unselected, treatment-seeking samples of children and adolescents [22,24]. However, knowledge on trauma characteristics, mental health, the need for treatment and the families’ further pathways through the healthcare system among a naturalistic sample [16,17] will provide valuable contributions to the development and implementation of treatment approaches for this vulnerable group. While for adult populations, few studies with unselected clinical samples are available [25,26], for children and adolescents, comparable data are scarce.

This clinical report intents to fill the gaps in the existing literature by presenting the approach and the data from the health care seeking sample of the OTC in Berlin, Germany, affiliated with the Department of Child Psychiatry and Psychotherapy. The first aim of this clinical report is to systematically describe the sample of the OTC with respect to sociodemographic variables, trauma-related and intervention-related variables. The second aim is to explore the range of trauma-related mental disorders across all age groups. The third aim is to conduct systematic dropout analyses followed by a presentation of treatment outcome and an analysis of its predictors. In line with the literature, we hypothesized that female gender, younger age, a longer time span untreated, the experience of sexual abuse and the experience of multiple traumatic events were associated with poor outcome.

## 2. Materials and Methods

### 2.1. Procedure in the OTC

The formal requirements for treatment in the OTC are child age <18 years, Berlin residency and the experience of interpersonal violence. All cases are assessed and treated by child and adolescent psychotherapists specialized in trauma-focused psychotherapy. With a contingent of up to 18 sessions, the OTC conducts a comprehensive psychological assessment, provides psychosocial support (e.g., a clearance of the situation, and the support of the family with formal steps), and first psychological interventions (e.g., stabilization, psychoeducation, provision of coping skills, parental counseling and, depending on the progress and stability, trauma exposure resp. trauma narratives). The intervention in the OTC follows an adaptive, tailored approach, mirroring the concept of trauma-informed care [22]. At the end of the treatment, individual recommendations for further interventions are stated and longer-term follow-up-intervention for the patients is initiated, if needed.

For this paper, data of all the cases who have consulted the clinic since its foundation are presented (April 2012–March 2020). Data are derived from the internal documentation and from the final letter, which is required for funding. The letter needs to include the anamnesis, results of the psychological assessment together with an evaluation whether or not the psychological symptoms are causally related to the experience of the traumatic event(s), descriptions of the interventions, of treatment outcome and, if applicable, a description of specific recommendations for further treatment.

The concomitant research of the OTC has been approved by the Ethics Committee of the Charité—Universitätsmedizin Berlin (number EA2/145/18, date of approval 30 July 2018).

### 2.2. Measures

#### 2.2.1. Sociodemographic and Trauma-Related Data

With the use of a structured questionnaire, sociodemographic data and descriptive data on the traumatic event (type of trauma, single vs. multiple events, offender, time span until first visit in the clinic) were collected. The types of traumatic events represented in the clinic are predefined by the Crime Victim Compensation Act and are categorized into sexual abuse, physical violence, witnessing violence, being victim of an attack, and other interpersonal trauma like psychological distress due to indirect involvement in violence, e.g., the information of a sudden death of a close person.

#### 2.2.2. Mental Disorders According to ICD-10, Axis 1

For each case, at the beginning of treatment, comprehensive clinical interviews are carried out in order to evaluate (1) the absence or presence of a mental disorder according to the ICD-10 classification system and (2) whether or not the symptoms were related to the traumatic event.

#### 2.2.3. Intervention-Related Data

We analyzed the number of sessions, the time range of the intervention and the recommendations concerning follow-up treatment on a descriptive level. For each case, the clinicians evaluated whether or not further follow-up intervention was required and if so, whether it should focus on the trauma, on other psychological or psychosocial problems or on a combination of both.

#### 2.2.4. Improvement of Psychological Symptoms

The measure for the improvement of psychological symptoms used in the OTC is derived from the standardized, therapist-reported documentation system for all patients in inpatient and outpatient treatment in the Department of Child and Adolescent Psychiatry. Thus, at the end of treatment in the OTC, the overall change of psychological symptoms was assessed in therapist report by the item “improvement of psychological symptoms”, rated on a standardized 5-point scale (fully improved, strongly improved, improved a little, no change, worsened). In logistic regression analyses, good outcome was defined as 1 = fully improved or clearly improved.

### 2.3. Data Analysis

Data were analyzed with SPSS, Version 25. Descriptive data analyses were used for the presentation of sample characteristics as well as trauma-related- and intervention-related characteristics. As the samples cover a broad age group, sensitivity analyses for age were conducted. For the drop out analyses, three groups (completers vs. referrals vs. decliners) were defined and compared on sociodemographic-, trauma- and intervention-related variables. In case of statistical trends (*p* < 0.10) on an overall level, post-hoc-analyses were conducted. Predictors for treatment outcome were first analyzed by univariate logistic regression, providing information on the importance of each predictor by itself. Following an explorative approach, we systematically tested sociodemographic predictors (child age, gender), trauma type (sexual abuse yes/no), other trauma-related characteristics (offender, multiple events yes/no), intervention-related data (number of sessions, time span until first visit) and baseline symptoms of posttraumatic stress (intrusions and avoidance scores derived from the CRIES-8 [27] as predictors. The significant predictors on univariate level were entered in a multivariate regression to gain a more comprehensive picture. For the regression analyses, only data of the completers were used.

## 3. Results

### 3.1. Participants

In total, 377 patients were treated in the OTC till 3/2020, with a mean age of M = 10.95 years (SD = 4.69, range 0.58–18.50 years). 80.3% of the sample was above the age of 6 years (0–5 years *n* = 74, 19.6%; 6–13 years *n* = 169, 44.8%; 14–18 years *n* = 134, 35.5%). 55.4% of the patients were girls. Table 1 summarizes trauma-related descriptive data of the sample. There were no statistically significant differences between the age groups in the trauma-related variables.

**Figure 1:**
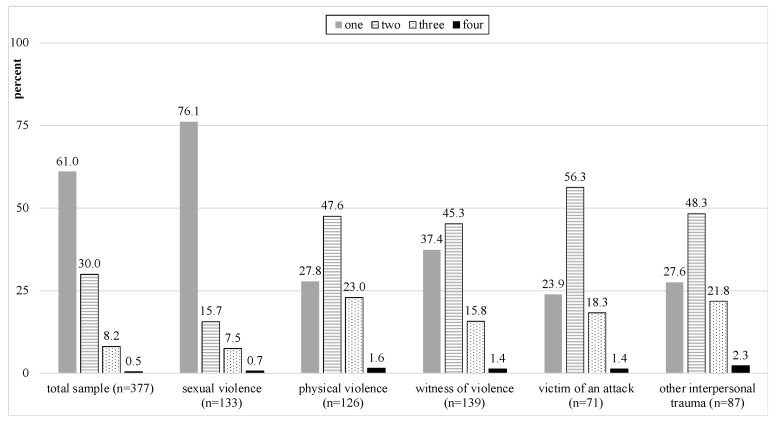
Co-occurrence between trauma types. The bars refer to the numbers of other trauma types present for each primary trauma type.

In the total sample, the majority presented with one (*n* = 230, 61.0%) or two different trauma types (*n* = 113, 30.0%). The rate of co-occurrence varied across the trauma types (see Figure 1). With the exception of sexual abuse, the co-occurrence of two different trauma types were the most frequent pattern.

### 3.2. Mental Disorders According to ICD-10

Structured anamnesis and psychiatric assessment provided an Axis 1-diagnosis according to ICD-10 in 310 cases (82.2%). The clear majority of diagnoses were trauma-related, i.e., the symptoms occurred after the traumatic event(s) (*n* = 288; 92.9%). In 22 cases (7.1%) only, the diagnosis was present already before the trauma. The onset of a broad range of mental disorders was observed. Adjustment disorders and PTSD were the primary trauma-related mental disorder in the majority of cases, across all age groups (81.9% of the total sample). In the age group 14–18 years, depressive disorders were the third most frequent primary trauma-related disorder; while in the age group 6–13 years, externalizing disorders were the third most frequent disorder and in the age group 0–5 years, emotional disorders, respectively. On average, comorbidity was relatively low, with 1.37 diagnoses per patient (SD = 0.70, Median = 1, Modus = 1; range 1–4). Table 2 summarizes the diagnoses on Axis 1 according to ICD-10.

### 3.3. Drop out Analyses

In total, 242 cases (64.2%) completed the treatment in the OTC (“completers“). 26 cases (6.9%) were referred to inpatient treatment due to the high severity of psychological symptoms (“referrals”). While in 13 cases (3.4%) organizational reasons (e.g., no Berlin residency) prohibited further treatment, 92 cases (24.4%) did not continue their sessions and were lost in follow-up (“decliners”). There was a trend that the time span between trauma and first visit differed between groups (F(2, 257) = 2.972, *p* = 0.053), with the highest time span until the first visit at the OTC for the referrals to inpatient treatment (M = 24.85, SD = 28.24) and the shortest for the completers (M = 13.67, SD = 18.56). There were significant differences between the three groups on the level of avoidance (F(2, 128) = 4.42, *p* = 0.014) and the total PTSD score at baseline (F(2, 140) = 3.909, *p* = 0.022) as measured with the CRIES-8 [27]. Post-Hoc tests showed significant higher avoidance scores in the decliners (M = 3.93, 0.88) than in the completers (M = 3.12, SD = 1.18; see Table 3). Concerning the duration of the intervention till drop out, decliners (M=2.72 months, SD = 4.13) and referrals to inpatient treatment (M = 3.85 months, SD = 4.76) showed a comparable time in intervention, but significantly shorter than the completers (M = 7.51 months, SD = 5.85, *p* = 0.003 and *p* < 0.001, respectively). The three groups did not differ with respect to gender (χ^2^(2) = 2.160, *p* = 0.340), age (F(2, 357) = 0.280, *p* = 0.756) nor trauma type (sexual violence (χ^2^(2) = 5.239, *p* = 0.073; physical violence (χ^2^(2) = 1.024, *p* = 0.599; witness of violence (χ^2^(2) = 1.183, *p* = 0.554; victim of an attack (χ^2^(2) = 1.211, *p* = 0.546). Table 3 summarizes the results of post-hoc tests for those variables with significant overall effects.

### 3.4. Intervention-Related Data

#### 3.4.1. Number of Sessions

Analyses on the completers revealed that on average, 10.18 (SD = 6.04) sessions were conducted (Median = 9, Modus = 18, range 1–18). While there were no age or gender effects, victims of sexual abuse received a significantly higher number of sessions than the other trauma types (M = 11.72, SD = 6.45 versus M = 9.05, SD = 5.55, *p* = 0.001). The average time span of intervention was 23.93 weeks (SD = 23.02, range 0–126 weeks, Median = 17 weeks). There were no differences for gender, age or trauma type for the time span of intervention.

#### 3.4.2. Recommendation for Further Treatment

Regarding the 239 completers, in about two thirds (*n* = 153, 64.0%), follow-up treatment was recommended. Outpatient psychotherapy (*n* = 113, 73.9%) was the most frequent recommendation at the end of the intervention, followed by recommendations for outpatient child psychiatry in 35 cases (22.9%), inpatient psychiatric treatment in one case (0.6%) and social measures for child protection in four cases (2.6%). For the clear majority (*n* = 142, 95.3%), further trauma-related psychotherapeutic intervention was recommended. Age groups did not differ in terms of recommendations (results not shown). The majority (69.4%) of the victims of sexual abuse needed further treatment, which was in contrast to victims of an attack (35.4% were recommended further treatment). For the other trauma types, about half of the cases were in need for further intervention (51.7–55.4%).

### 3.5. Analyses of Treatment Outcome

For the cases completing the intervention (*n* = 235), about half of the cases showed good outcome (*n* = 121, 51.5%), defined as full improvement (*n* = 28), or strong improvement (*n* = 93) of psychological symptoms. The other large part improved a little (*n* = 94; 40.0%). For 19 cases, no change was observed (8.1%) and in one case, worsening of psychological symptoms was stated (0.4%). There were no age or gender effects in the rating (results not shown).

Table 4 summarizes the significant predictors derived from the univariate regression analyses on good treatment outcome (coded with 1 = strong or full improvement of psychological symptoms). Female gender, victims of multiple traumatic events and victims of sexual violence as well as the cases with a higher number of intervention sessions were at risk for poor treatment outcome. The other sociodemographic, trauma-or intervention-related predictors were not significantly related with the outcome (results not shown).

Additionally. in multivariate regression, the presence of multiple traumatic events at presentation (B = −0.823, SE = 0.313, *p* = 0.009, OR = 0.439, 95% CI 0.238–0.811) and, with a weaker effect, a higher number of sessions (B = −0.070, SE = 0.025, *p* = 0.006, OR = 0.933, 95% CI 0.887–0.980) were inversely related with good treatment outcome.

## 4. Discussion

This report presented the interventional approach and clinical data from the OTC for child and adolescent victims of interpersonal violence. In this paper, we aimed to (1) provide a comprehensive account of trauma-specific characteristics of the treatment-seeking sample, (2) assess the onset and rates of trauma-related mental disorders, (3) describe intervention-related data and (4) evaluate in how far improvements in psychological symptoms can be achieved within the scope of the first-care, short-term naturalistic intervention. Furthermore, factors related to drop out were explored.

Summing up the descriptive analyses, the trauma types were equally distributed across the age groups, an average co-occurrence of one to two trauma types was observed and over one third of the sample experienced multiple events within one trauma type. A high proportion was acutely presenting at the clinic within the first two weeks after trauma. In the majority of cases, the offenders were known from the personal environment, which implies that within this first-care setting, additional measures for safety and child protection were regularly warranted.

The second aim was to give an account on the mental health in the clinical sample. In total, 82.2% were diagnosed with a mental disorder, and of these, in 92.9%, the onset was related to the experience of the trauma. Adjustment disorders and PTSD were the most prevalent diagnoses, in line with epidemiological data [1,6,28]. While adjustment disorders were more frequently diagnosed up to the age of 13 years, the majority of adolescents suffered from PTSD. School-aged children additionally showed externalizing symptoms and onset of enuresis or encopresis while the preschoolers showed a higher rate of childhood emotional disorders like separation anxiety. The broad range of clinical diagnoses and especially the higher variety of comorbid diagnoses like substance abuse and personality disorders in the adolescent subgroup mirror the relevance of transdiagnostic approaches in trauma-focused care [29] and the need for a broad psychiatric assessment. The complexity of the acutely presenting cases also underline the need for an adapted clinical approach with an increased share of psychosocial and psychological stabilization [30].

The third aim was to explore intervention-related data and treatment outcome. The average treatment duration, independent of age, gender or trauma type, covered approximately four to six months and on average, ten sessions were provided. The regression analyses aimed to get a better picture of the characteristics related to treatment outcome. Results replicated findings in the literature, as in our sample, girls [7,8,9] and victims of sexual abuse [4,11] were at risk for poor treatment outcome. Beyond all other predictors, children and adolescents with a history of multiple traumatic events, independent of the type of trauma, were at highest risk for poor improvement of psychological symptoms, which adds to previous findings as well [12,31]. Noteworthy, in this report, the outcomes only refer to therapists’ rating at the end of the intervention. While this measure is an established part of the internal documentation and quality assurance in the university clinic, the one-item measure only captures improvement on an overall level, limiting generalizability for further, more specific psychological symptoms. Further, only therapist ratings are collected and patient-reported data are currently not available. Therefore, for further proceedings of the OTC’s and other clinical services’ concomitant research, the use of validated and more differentiated measures for treatment outcome, both in therapist and in patient-report need to be implemented. This will increase validity and generalizability, and will facilitate future replication of our findings.

Dropout analyses indicated that the majority of cases regularly finished their treatment in the OTC. At 24%, the rate of decliners in our naturalistic setting was comparable to that of completers in large randomized-controlled trials for the evaluation of trauma-focused cognitive behavioral therapy [32,33]. However and noteworthy, the context conditions (e.g., naturalistic clinical setting vs. standardized research setting; treatment seeking sample vs. participation in a clinical trial) are differing to a high degree, therefore comparisons are limited. Further naturalistic studies are warranted in order to gain more insight into attrition and more knowledge on how to best prevent early drop out. In our sample, the cases who dropped out of treatment showed the highest level of avoidance symptoms. This highlights the relevance of early screening particularly for avoidance symptoms, which will provide valuable information and individual target points for preventive actions. Thus, the screening needs to be followed by an early provision of strategies to target avoidance, e.g., symptom and trigger monitoring and provision of coping skills. These measures may help to identify cases at risk at an early stage and to reduce dropout from trauma-focused intervention. Besides avoidance symptoms, the time span between the traumatic event and presentation in the clinic emerged as relevant factor, too: the decliners showed a significantly longer time span till their first visit than the completers. In order to reduce the time span untreated, barriers need to be addressed. This calls for better access to care [18], increased availability of clinics [19,20] and trauma-informed approaches [22], in order to reach out to those in need for treatment. 

The overlap between trauma types was comparable with the rate reported for other community samples [31,32]. While this is a common phenomenon, conclusions of the trauma type-specific analyses in this paper are limited. Nevertheless, for victims of sexual abuse a more distinct pattern was observed on a descriptive level. While the rate of co-occurrence with other trauma types was comparably low, a higher number of sessions was required than for the other trauma types and the majority was in need for follow-up-intervention. The data therefore suggest that a tailored approach for the specific requirement of victims of sexual abuse might be warranted, e.g., by offering an extended quota of sessions. 

The data from this report entail public health relevancy. First, the need for a specialized and accessible outpatient clinic for traumatized children and adolescents is evidenced by the high number of patients since the foundation of the trauma clinic, with about one new referral per week. Second, by the accessibility of the clinic, the burdened cases did receive an early trauma-informed and individualized intervention, contrasting the otherwise high probability that they were left untreated and not connected to any health services, for a longer time span at least [19,20]. Therefore trauma-informed facilities such as the OTC can play an important bridging role between onset of the trauma and the access to early intervention and the mental health care system: For one third of the patients, the provision of an initial clinical assessment, clearing and stabilization at the OTC led to reduction of psychological distress to the extent that no further follow-up treatment was required (and thereby helping to reduce the pressure on health care overload and costs). For the other two thirds, targeted follow-up treatment could be recommended and initiated by the clinic to contribute to sustained care. Further data on mental health care use and longer-term outcome are not yet available; however, a 3–12-months follow-up study is currently running.

In conclusion, early trauma-informed treatments, like described in the literature [22] and like the early intervention approach of the OTC described in this paper, are able to provide comprehensive and specialized support the families during early stages after the traumatic event; this may mitigate the risk for persistence of symptoms in the vulnerable group of children and adolescents.

Nationwide policies within the health care system need to ensure that trauma care for this vulnerable group is available and accessible [17] in order to facilitate assessment and intervention. Furthermore, knowledge on treatment centers’ procedures and on their implementation of care will help to disseminate treatment approaches, which is especially warranted in the area of child and adolescent trauma, where a lack of treatment has been observed [19]. Therefore, enabling fast referral to specialized trauma-informed treatment centers like the OTC, and building up regional networks of trauma-informed care to foster direct follow-up treatment for the affected families are the key efforts which must urgently and widely be undertaken in clinical practice. Thus, we call other psychiatric and pediatric clinics to adapt this early treatment approach for the sake of improving and expanding the care for traumatized children and adolescents.

## Figures and Tables

**Table 1:** Descriptive data in relation to the traumatic event.

	Total Sample *n* = 377		Age Groups					
			0–5 Years		6–13 Years		14–18 Years	
			*n* = 74		*n* = 169		*n* = 134	
	*n*	(%)	*n*	(%)	*n*	(%)	*n*	(%)
Type of traumatic event ^†^								
Sexual abuse	133	35.50%	15	23.40%	56	33.10%	62	46.30%
Physical violence	126	33.60%	31	48.40%	45	26.60%	50	37.30%
Witness of violence	139	37.10%	43	67.20%	71	42.00%	25	18.70%
Interpersonal attack	71	18.90%	5	7.80%	21	12.40%	45	33.60%
Other ^‡^	87	23.30%	18	28.10%	41	24.30%	28	20.90%
Frequency of trauma ^§^								
Single event	232	61.50%	27	42.20%	97	57.40%	108	80.60%
Multiple events	142	37.70%	45	70.30%	71	42.00%	26	19.40%
Offender								
Close environment	179	47.50%	60	93.80%	88	52.10%	31	23.10%
Broad environment	59	15.60%	4	6.30%	26	15.40%	29	21.60%
Unknown person	130	34.50%	7	10.90%	52	30.80%	71	53.00%
Unidentified	9	2.40%	3	4.70%	3	1.80%	3	2.20%
Time span between trauma and first visit ^¶^								
*M* (SD)	16.22 (23.79)		14.40(18.5)		14.03(17.7)		19.14(29.97)	
Median	6		7.14		6		5.5	
Modus	1.43		1.57		0.57		1.29	
range	0.14–178.9		1–71.43		0.29–72.14		0.14–178.86	

Notes. ^†^ multiple answers possible; ^‡^ e.g., psychological distress due to indirect involvement in violence, e.g., the information of a sudden death of a close person; ^§^ in 3 cases data were missing; ^¶^ time span between trauma and first visit in weeks.

**Table 2:** Mental disorders in relation to the traumatic event on axis-1 according to ICD-10, summarized in categories of disorders, for the total sample and the age groups.

	Total Sample *n* = 278		Age 0–5 Years *n* = 49		Age 6–13 Years *n* = 121		Age 14–18 Years *n* = 108	
	*n*	(%)	*n*	(%)	*n*	(%)	*n*	(%)
**Stress-related disorders**								
PTSD (F43.1)	115	(41.7%)	12	(24.5%)	49	(40.5%)	54	(50.0%)
Adjustment disorder (F43.2x)	111	(40.2%)	17	(34.7%)	57	(47.1%)	37	(34.3%)
Acute reaction disorder (F43.0)	7	(2.5%)	1	(2.0%)	0	(0.0%)	6	(5.6%)
**Internalizing disorders**								
Depressive disorders (F32.x, F33.x, F34.x)	28	(10.1%)	0	(0.0%)	11	(9.1%)	17	(15.7%)
Anxiety disorder (F40.x, F41.x)	4	(1.4%)	0	(0.0%)	1	(0.8%)	3	(2.8%)
Emotional disorders (F93.x)	19	(6.9%)	12	(24.5%)	6	(5.0%)	1	(0.9%)
**Externalizing disorders**								
ADHD (F90.x)	15	(5.4%)	4	(8.2%)	11	(9.1%)	0	(0,0%)
Conduct disorder (F91.x)	19	(6.9%)	4	(8.2%)	11	(9.1%)	4	(3.7%)
**Other disorders**								
Psychoactive substance use (F1x.x)	9	(3.3%)	0	(0.0%)	3	(2.5%)	6	(5.6%)
Obsessive compulsive disorder (F42.x)	3	(1.1%)	0	(0.0%)	1	(0.8%)	2	(1.9%)
Dissociation disorder (F44.x)	3	(1.1%)	0	(0.0%)	1	(0.8%)	2	(1.9%)
Somatoform disorder (F45.x)	5	(1.8%)	0	(0.0%)	1	(0.8%)	4	(3.7%)
Eating disorder (F50.x, F51.x)	4	(1.4%)	0	(0.0%)	0	(0.0%)	4	(3.7%)
Personality disorder (F60.x)	10	(3.6%)	0	(0.0%)	1	(0.8%)	9	(8.3%)
Attachment disorder (F94.1, F94.2)	7	(2.5%)	3	(6.1%)	4	(3.3%)	0	(0.0%)
Other disorder of social functions (F95)	2	(0.7%)	0	(0.0%)	2	(1.7%)	0	(0.0%)
Enuresis/Encopresis (F98.0, F98.1)	11	(4.0%)	2	(4.1%)	9	(7.4%)	0	(0.0%)
Other behavioral or emotional disorder (F98.8)	6	(2.2%)	4	(8.2%)	1	(0.8%)	1	(0.9%)

Notes. *n* in the table refers to the number of cases with the specific diagnosis, %-score refers to the percentage within the total sample resp. within the age group for each category of diagnoses. The highest proportion of a specific disorder across the three age groups is marked in bold. Table includes comorbidities.

**Table 3:** Results of posthoc-tests for variables with a significant overall effect in the drop-out analyses.

Variable	Subgroup †		*n*	*M*	SD	Post Hoc 1 vs. 2			Post Hoc 1 vs. 3			Post Hoc 2 vs. 3		
						*MD*	SE	95% CI	*MD*	SE	95% CI	*MD*	SE	95% CI
Time till first visit in weeks	1	Completers	187	13.67	18.56	11.18	5.78	−25.10–2.74	−5.93	3.37	−14.05–2.19	5.24	6.23	−9.78–20.27
	2	Referrals	18	24.85	28.24									
	3	Decliners	65	19.60	32.78									
Number of sessions	1	Completers	242	10.15	6.04	4.65 ***	**1.12**	**1.96–7.34**	5.75 ***	**0.67**	**4.15–7.36**	1.10	1.21	−1.80–4.00
	2	Referrals	26	5.50	3.46									
	3	Decliners	91	4.40	3.93									
Time in treatment (months)	1	Completers	243	7.51	5.85	3.67 **	**1.11**	**0.99–6.34**	4.79 ***	**0.66**	**3.21–6.38**	1.12	1.20	−1.75–4.01
	2	Referrals	26	3.84	4.76									
	3	Decliners	92	2.72	4.13									
Avoidance symptoms ‡	1	Completers	96	12.50	5.57	−2.68	1.66	−6.70–1.34	−3.21 *	**1.19**	**−6.09–−0.33**	−0.53	1.89	−5.12–4.07
	2	Referrals	11	15.18	4.22									
	3	Decliners	24	15.71	3.85									
PTSD total score ‡	1	Completers	109	23.16	9.98	−6.48	2.97	−13.67–0.71	−4.32	2.15	−9.53–0.89	2.15	3.44	−6.17–10.49
	2	Referrals	11	29.64	5.78									
	3	Decliners	23	27.48	7.49									

Notes. † Referrals include the cases with high severity, referred to inpatient treatment; decliner include the cases who dropped out of treatment; ‡ as measured with CRIES-8 at baseline [27]; MD = mean difference, SE = standard error, CI = confidence interval for MD. Significant differences in bold. * *p* < 0.05, ** *p* < 0.01, *** *p* < 0.001.

**Table 4:** Summary of significant predictions in univariate logistic regression on good treatment outcome, defined as “improvement of psychological symptoms” at the end of the intervention (coded with 1, *n* = 121).

	Improvement of Psychological Symptoms				
	B	SE	*p*	OR	95% CI OR
**Sociodemographic predictors**					
Female gender	−0.544	0.266	0.041	0.581	0.345–0.978
**Trauma-related predictors**					
Multiple traumatic events	−0.976	0.282	0.001	0.377	0.217– 0.655
Sexual abuse	−0.852	0.287	0.003	0.427	0.243– 0.750
**Intervention-related predictors**					
Number of sessions	−0.069	0.023	0.002	0.934	0.893–0.976

Notes. The categorial predictors female gender, multiple traumatic events and sexual abuse all included with 1 = yes in the regression analyses.

## Data Availability

The data that support the findings of this study are available upon reasonable request from the corresponding author. The data are not publicly available due to privacy or ethical restrictions.
